# A comparative Raman study between *PrMnO*_3_, *NdMnO*_3_, *TbMnO*_3_ and *DyMnO*_3_

**DOI:** 10.1038/s41598-017-12714-8

**Published:** 2017-10-23

**Authors:** Sabeur Mansouri, Serge Jandl, Alexander Mukhin, Vsevolod Yu Ivanov, Anatoly Balbashov

**Affiliations:** 1 Université de Sherbrooke, Regroupement Québécois sur les Matériaux de Pointe et Institut Quantique, Département de Physique, Sherbrooke, J1K 2R1 Canada; 2 Prokhorov General Physics Institute of the Russian Academy of Sciences, 38 Vavilov St., Moscow, 119991 Russia; 3 Moscow Power Engineering Institute, 14 Krasnokazarmennaya St., Moscow, 105835 Russia

## Abstract

In this paper, we present a detailed Raman study of the non-multiferroic compounds *PrMnO*_3_ and *NdMnO*_3_ and the multiferroic compounds *TbMnO*_3_ and *DyMnO*_3_ as a function of temperature and magnetic field. All studied systems show anomalous phonon shifts close to the Néel transition *T*_*N*_. In *PrMnO*_3_ and *NdMnO*_3_, the frequency softenings are partly attributed to an orbital-spin-phonon coupling whereas in *TbMnO*_3_ and *DyMnO*_3_, the relatively weak frequency shifts are rather attributed to an expansion of the *Mn*−*O* bond lengths. On the other hand, the frequencies of *TbMnO*_3_ phonons are shifted as a function of magnetic field, while those of *PrMnO*_3_ remain unaffected. These frequency shifts are interpreted in terms of local oxygen rearrangements under magnetic field that could play an important role in the multiferroicity of *TbMnO*_3_ and *DyMnO*_3_.

## Introduction

In the last decade, the study of the perovskite manganites *RMnO*_3_ (*R* = lanthanides, *Y* and *Bi*) have attracted a considerable interest because of their fascinating properties such as colossal magnetoelectric and magnetocaloric effects^1,2^. Their physical properties are determined by a delicate interplay of charge, spin, orbital, and lattice degrees of freedom^3–5^. The study of the coupling between the lattice and the magnetic properties of these compounds represents an interesting starting point to understand the microscopic parameters controlling their magnetoelectric effects at low temperature. The *RMnO*_3_ with larger ionic radius (*R* = *La*, *Pr*, *Nd*,… and *Dy*) compounds crystallise in the orthorhombic structure with a space group *Pbnm*, whereas the compounds with smaller ionic radius (*R* = *Dy*, *Ho*, *Er* and *Y*) can be obtained either in the orthorhombic or the hexagonal structure with a space group *P*6_3_ *cm* ^6,7^. In the orthorhombic compounds the *Mn* ion has the ${t}_{2g}^{3}{e}_{g}^{1}$ electronic configuration. The ${e}_{g}^{1}$ orbital degeneracy is lifted by the Jahn-Teller distortion^5,8^. These compounds exhibit an orbital ordering at high temperature ${T}_{OO}\sim 780-1500\,K$ ^9–11^. Below *T*_*OO*_, the orbital degree of freedom is spontaneously frozen by the real-space *C*–type ordering of the *e*_*g*_ orbitals accompanied by the development of a static Jahn-Teller lattice distortion of the *MnO*_6_ octahedra^3,4^. Many previous works have suggested a strong coupling between the spin and the orbital degrees of freedom in the *RMnO*_3_ systems^3,8,12–14^. Murakami *et al*.^8^ have reported an experimental evidence of the orbital ordering in *LaMnO*_3_ by measuring the (3,0,0) reflection intensity of resonant x-ray scattering^8^. They have found that its integrated intensity, which is related to the order parameter of the orbital ordering, becomes constant below the magnetic ordering temperature *T*_*N*_ ≈ 140 *K* and decreases above it, which suggests that the spin and the orbital orders are intercoupled. The orbital ordering temperature in *RMnO*_3_ is significantly enhanced by increasing the *GdFeO*_3_-type lattice distortion with decreasing the ionic radius of *R* (*r*_*R*_)^3,11^. These lattice distortions increase the orbital ordering frustration and provoke a change of the lattice parameters as shown by x-ray and neutron diffraction measurements^15,16^ with a sharp fall in the ordering temperature of the *Mn* spins^3^. For example, *PrMnO*_3_ (*NdMnO*_3_) exhibits an *A*-type antiferromagnetic magnetic structure at *T*_*N*_ ≈ 100 *K* (*T*_*N*_ ≈ 80 *K*) in which the spins are aligned ferromagnetically in the basal plane (*xz*) and antiferromagnetically along the perpendicular direction (*y*)^17,18^. In more distorted compounds (*R* = *Dy*, *Tb* and *Gd*) the spin structure becomes sinusoidally modulated in the *ab*–plane below 39–43 *K* and spirally modulated below 18-17 *K* ^19^. This last transition coincides with the appearance of a spontaneous electric polarization *P*_*S*_ parallel to the *c*–axis^1,20^. In these spin-spiral systems the magnetic field is able to flip their electric polarization from *c* to *a*–axis.

Raman scattering spectroscopy is a powerful tool to investigate the driving force of the interplay of charge, spin, orbital and lattice degrees of freedom^21–25^. The Raman spectra of *RMnO*_3_ orthorhombic compounds have been previous studied^21,22,26^. In particular, they have explained the frequency softening of the 490 *cm* ^−1^ and 600 *cm* ^−1^ modes below *T*_*N*_ in terms of spin-phonon coupling caused by the phonon modulation of the superexchange integral^21,22,26^. Recently, Xu *et al*.^27^ have attributed the softening behavior of the two excitations at 490 *cm* ^−1^ and 610 *cm* ^−1^ of *LaMnO*_3_ to an orbital-spin-phonon (OSP) coupling rather than limited to a spin-phonon coupling only. The verification of the suggested theoretical models requires more studies of the effects of magnetic and orbital orderings on the phonon frequencies in the *RMnO*_3_ compounds.

In this paper, we investigate the Raman-active phonons in *PrMnO*_3_, *NdMnO*_3_, *TbMnO*_3_ and *DyMnO*_3_ compounds as a function of temperature and magnetic field. The objectives are (i) to study and compare the Raman response of the non-multiferroic systems *PrMnO*_3_ and *NdMnO*_3_ to those of the multiferroic compounds *TbMnO*_3_ and *DyMnO*_3_, (ii) to study the effect of a magnetic field on the Raman-active phonons in the representative compounds *PrMnO*_3_ and *TbMnO*_3_ at low temperature.

## Results and Discussion

Based on group-theory, the orthorhombic *RMnO*_3_ (space group *Pbnm*) has twenty-four Raman-active modes (7*A*_*g*_ + 7*B*_1*g*_ + 5*B*_2*g*_ + 5*B*_3*g*_)^28^. The *RMnO*_3_ unit cell contains two *MnO*_6_ octahedra along the *y*–direction with two apical *O*2 and four equatorial *O*1 oxygen ions at the summits. The *Mn* − *O*2 bond is along the *y*–axis while the *Mn* − *O*1 bonds are in the *xz* plane (*ab*–plane).

**Figure 1:**
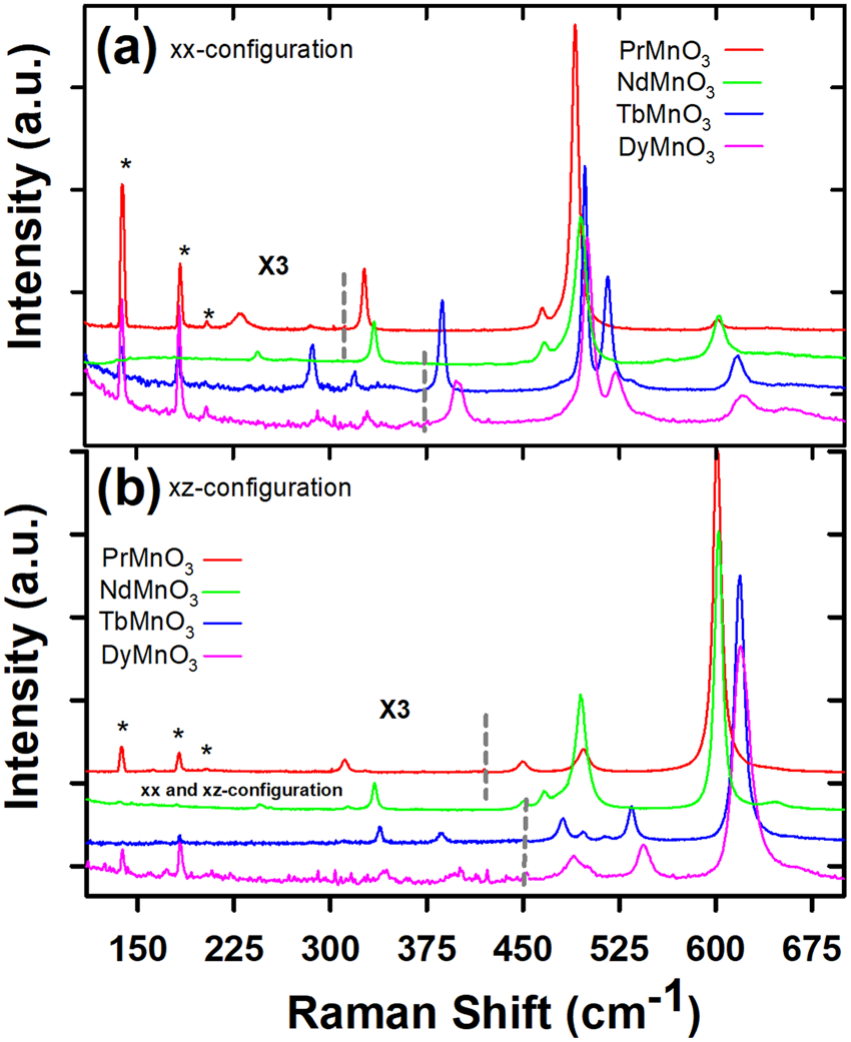
The polarized Raman spectra of *PrMnO*_3_, *NdMnO*_3_, *TbMnO*_3_ and *DyMnO*_3_ in the *xx* (**a**) and *xz* (**b**) configurations at *T* = 5 *K*. (★) indicates plasma lines.

Figure 1 shows the polarized Raman spectra of *RMnO*_3_ (*R* = *Pr*, *Nd*, *Tb* and *Dy*) in the *xx* (*aa*) and *xz* (*ab*) configurations at 5 *K*. The typical phonons associated with the orthorhombic *RMnO*_3_ manganites are observed^21,22^. For both configurations, six *A*_*g*_ and six *B*_1*g*_ Raman-active phonons are detected. The selection rules are well respected and the phonon linewidths are close to 3–7 *cm* ^−1^ attesting the high crystalline quality of the samples. With decreasing temperature, no additional modes appear indicating a structural phase stability of the *RMnO*_3_ compounds. The frequencies of the *A*_*g*_ and *B*_1*g*_ Raman active phonons of *PrMnO*_3_, *NdMnO*_3_, *TbMnO*_3_ and *DyMnO*_3_ are reported in Table 1. Their vibrational characters are assigned in agreement with previous Raman measurements^29^. With decreasing the ionic radius of *R* (*r*_*Pr*_ > *r*_*Nd*_ > *r*_*Tb*_ > *r*_*Dy*_) most phonon frequencies shift towards higher values. Simultaneously, there is a transfer of intensity between the two high-frequency *A*_*g*_ phonons close to 500 *cm* ^−1^ due to lattice distortions induced by the ionic radius size that mixes the symmetry characters of some phonon excitations^21^. Here we focus on the microscopic mechanisms and the theoretical models required to explain the phonon frequency shifts and how they differ in the multiferroic *TbMnO*_3_ and *DyMnO*_3_ compounds as compared to the non-multiferroic compounds *PrMnO*_3_ and *NdMnO*_3_.

**Table 1:** Frequencies (*cm* ^−1^) of the Raman-active modes in *PrMnO*_3_, *NdMnO*_3_, *TbMnO*_3_ and *DyMnO*_3_ at 5 K.

Modes	Atomic displacements^21,22,28^	*PrMnO*_3_	*NdMnO*_3_	*TbMnO*_3_	*DyMnO*_3_
*A*_*g*_	*Ag*				
(6)	*R* and *O*2 displacements	147	146	146	143
(2)	in-phase *MnO*_6_ *y* rotation	228	245	283	288
(7)	*O*1(*x*)	282	289	317	325
(4)	out of phase *MnO*_6_ *x* rotations	325	334	385	392
(1)	*O*2 antistretching	463	467	495	494
(3)	*MnO*6 bending	490	494	513	516
*B*_1*g*_	*B*1*g*				
(6)	*R*, *O*2 and *O*1 displacements	160	161	164	169
(4)	*R* and *O*2 displacements	208	210	310	312
(7)	*O*1(*z*)	309	313	338	338
(3)	out-of-phase *MnO*6 bending	448	450	480	481
(2)	in-phase *O*2 scissorslike	496	502	534	536
(1)	in plane *O*2 stretching	600	602	615	612

**Figure 2:**
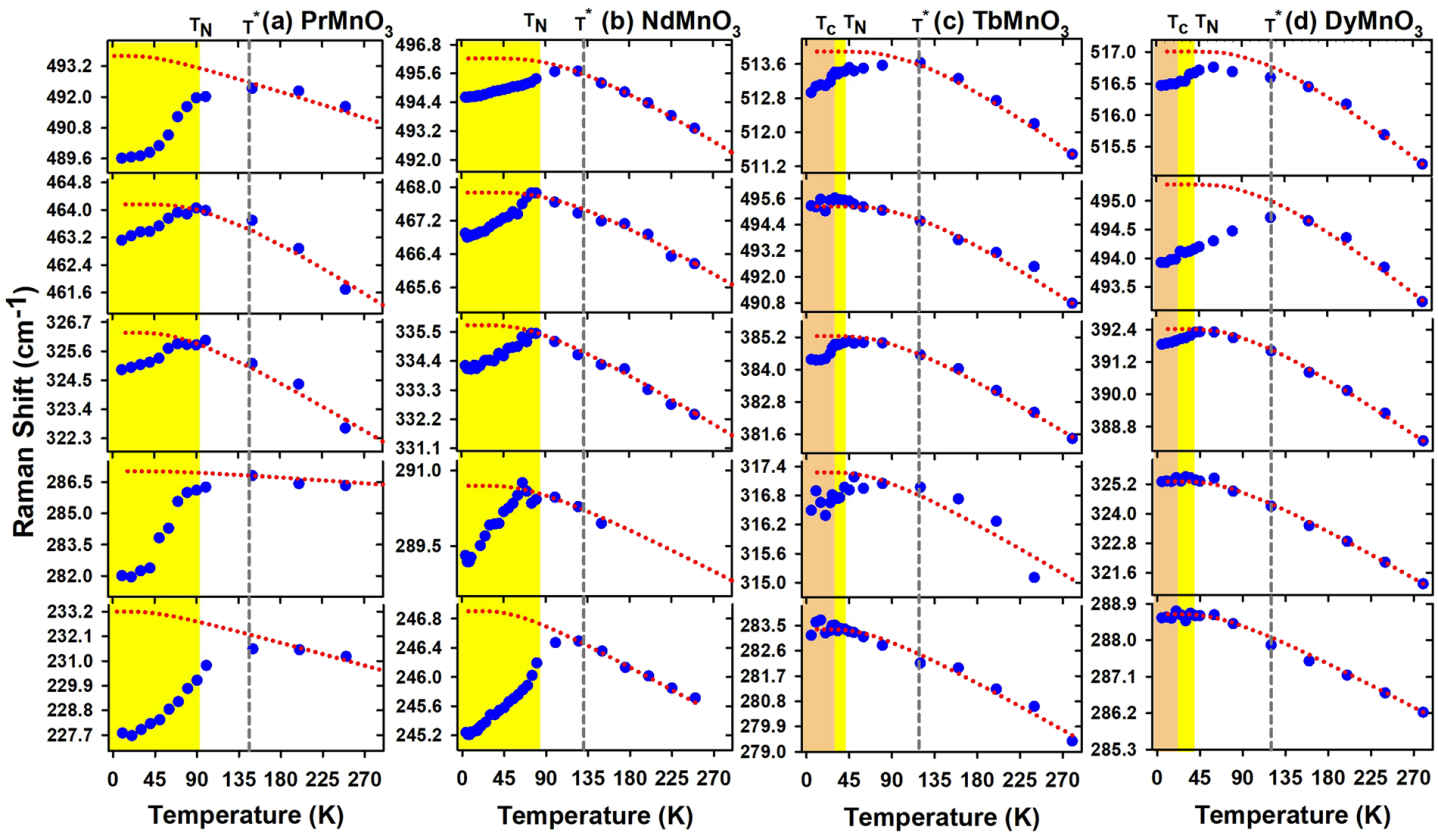
Temperature dependence of the *A*_*g*_ phonon frequencies of *PrMnO*_3_, *NdMnO*_3_, *TbMnO*_3_ and *DyMnO*_3_. Dotted lines correspond to the anharmonic behaviour.

**Figure 3:**
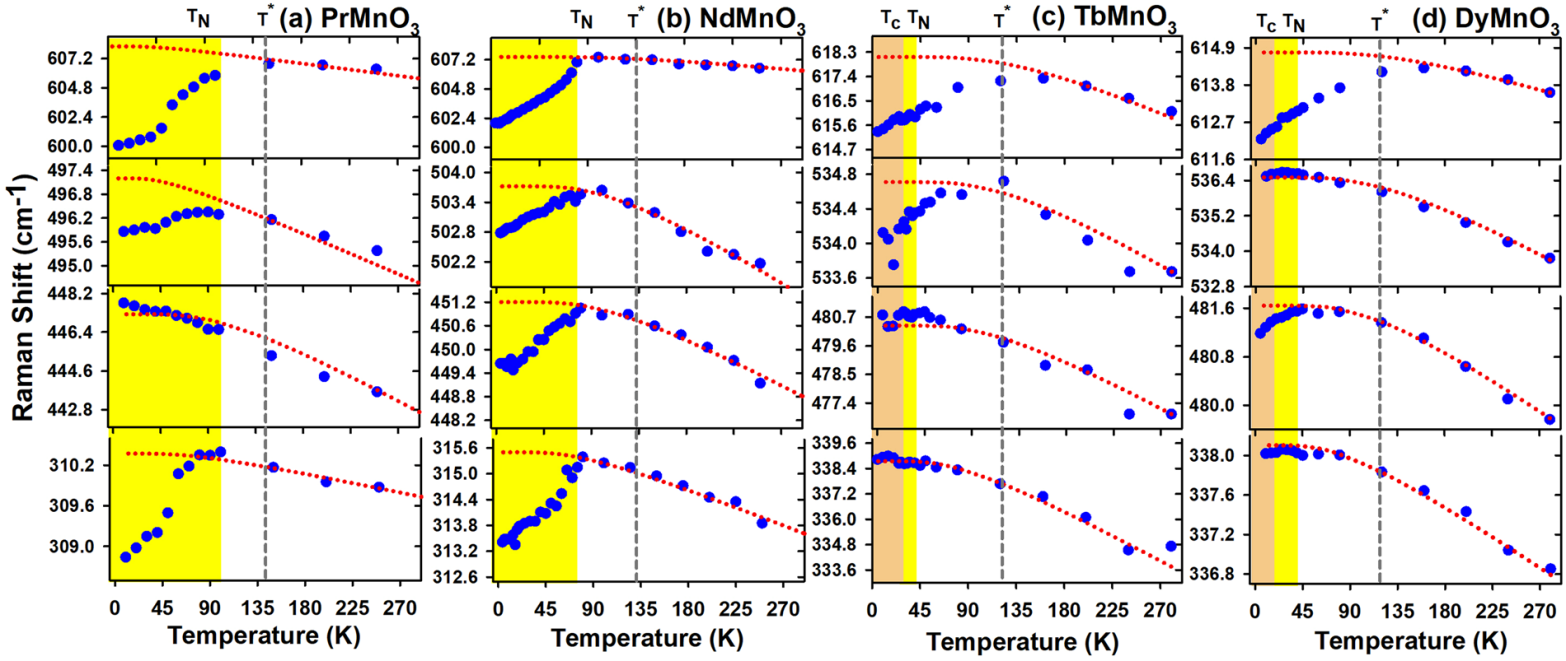
Temperature dependence of the *B*_1*g*_ phonon frequencies of *PrMnO*_3_, *NdMnO*_3_, *TbMnO*_3_ and *DyMnO*_3_. Dotted lines correspond to the anharmonic behaviour.

Figures 2 and 3 respectively show the temperature dependences of the *A*_*g*_ and *B*_1*g*_ phonon frequencies for *PrMnO*_3_ (a), *NdMnO*_3_ (b) *TbMnO*_3_ (c) and *DyMnO*_3_ (d). Between 300 *K* and *T* ^★^, the temperature evolutions of the different *RMnO*_3_ phonon frequencies are very similar: they harden with decreasing temperature. This frequency hardening is due to the anharmonic effect (dashed line) which can be simulated by the following formula: $\omega (T)={\omega }_{0}-C(1+\frac{2}{{e}^{x}-1})$, where $x=\frac{\hslash {\omega }_{0}}{2{K}_{B}T}$, *ω*_0_ and *C* are adjustable parameters. For *PrMnO*_3_ (*NdMnO*_3_), the frequencies of the most detected modes soften significantly below ${T}_{N}\sim 100\,K$ (${T}_{N}\sim 80\,K$). Remarkably, the frequencies of *A*_*g*_(2) at 228 *cm* ^−1^ (245 *cm* ^−1^), *A*_*g*_(3) at 490 *cm* ^−1^ (494 *cm* ^−1^) and *B*_1*g*_(1) at 600 *cm* ^−1^ (602 *cm* ^−1^) phonons soften by 1.3, 0.5, and 1.0 % (0.7, 0.4 and 0.8 %) respectively below *T* ^*N*^. For *TbMnO*_3_, the frequencies of the phonons *A*_*g*_(3) at 513 *cm* ^−1^, *B*_1*g*_(2) at 534 *cm* ^−1^ and *B*_1*g*_(1) at 615 *cm* ^−1^ deviate from the regular anharmonic behavior and soften unexpectedly below 130 *K*. These frequency softenings are slightly pronounced below *T*_*N*_ and much weaker as compared to their corresponding in *PrMnO*_3_ and *NdMnO*_3_. For example, the frequency softening of the *B*_1*g*_(1) mode is 2 *cm* ^−1^ for *TbMnO*_3_ while it is 6 *cm* ^−1^ for *PrMnO*_3_. Also, frequencies of the *A*_*g*_(4) mode and *A*_*g*_(7) mode show a weak energy softening about 0.6 *cm* ^−1^ below ${T}_{N}\sim 40\,K$, whereas those of *A*_*g*_(1), *A*_*g*_(2), *B*_1*g*_(3) and *B*_1*g*_(7) reproduce an anharmonic behavior. For *DyMnO*_3_, the temperature behaviors of the phonon frequencies are similar to those of *TbMnO*_3_. Interestingly, the unexpected frequency softenings of the Jahn-Teller phonons (*A*_*g*_(3) et *B*_1*g*_(1)) start well above *T*_*N*_ and are slightly enhanced below *T*_*N*_.

### Analysis of temperature effects

The frequency softening of Raman phonons below *T*_*N*_ in the *A*–type *RMnO*_3_ compounds was previously associated to a spin-phonon coupling^22,26^. Considering the nearest neighbours of the *Mn* ^3+^ ions, Granado *et al*. have shown that the Raman frequency shift is proportional to the square of the *Mn* ^3+^ ferromagnetic sublattice magnetization, [*M*(*T*)]^2^ as defined in eq. (1).

1 $${({\rm{\Delta }}{\omega }_{\alpha })}_{s-ph}\approx -\frac{2}{{\mu }_{\alpha }{\omega }_{0}}(\frac{{\partial }^{2}{J}_{xz}}{\partial {u}_{O1}^{2}}-\frac{1}{2}\frac{{\partial }^{2}{J}_{y}}{\partial {u}_{O2}^{2}}){[\frac{M(T)}{4{\mu }_{B}}]}^{2}$$

where *J*_*xz*_ and *J*_*y*_ are the exchange constants in the equatorial plane and along the apical axis respectively and *u*_*k*_ is the displacement vector from the equilibrium positions of the *k* ^*th*^ *O* ^2−^ ion. Xu *et al*.^27^ have revised the spin-phonon coupling model and have developed a theoretical model that takes into account the contribution of the orbital ordering. The total frequency shift Δ*ω*_*OSP*_(*T*) is written as a function of the effective force constant of the spin-phonon coupling (first term) and the effective force constant of orbital-spin-phonon (second term).

2 $${\rm{\Delta }}{\omega }_{OSP}(T)\approx -\frac{1}{2{\mu }_{\alpha }{\omega }_{0}}\sum _{i,j}(\frac{{\partial }^{2}{J}_{1}}{\partial {u}^{2}}{S}_{i}{S}_{j}+4\frac{{\partial }^{2}{J}_{3}}{\partial {u}^{2}}{S}_{i}{S}_{j}{\tau }_{i}{\tau }_{j})$$

where *τ*_*i*_ *τ*_*j*_ are the orbital correlation operators and *S*_*i*_ *S*_*j*_ are the spin correlation functions for the *i* and *j* Mn^3+^ ions. Using the multivariable Taylor expansion ∑_*i*,*j*_(*S*_*i*_ *S*_*j*_ *τ*_*i*_ *τ*_*j*_) ≈ ∑_*i*,*j*_(〈*S*_*i*_ *S*_*j*_〉〈*τ*_*i*_ *τ*_*j*_〉 + 〈*S*_*i*_ *S*_*j*_〉*δτ* + 〈*τ*_*i*_ *τ*_*j*_〉*δS*), where $\delta \tau \equiv {\tau }_{i}{\tau }_{j}-\langle {\tau }_{i}{\tau }_{j}\rangle {|}_{{S}_{i}{S}_{j}=\langle {S}_{i}{S}_{j}\rangle }$ and $\delta S\equiv {S}_{i}{S}_{j}-\langle {S}_{i}{S}_{j}\rangle {|}_{{\tau }_{i}{\tau }_{j}=\langle {\tau }_{i}{\tau }_{j}\rangle }$, one can establish the expression of the frequency softening of some Raman active modes^27^. The frequency shift Δ*ω*(*T*) of the in-phase stretching mode at $\sim 600\,c{m}^{-1}$ becomes

3 $$\begin{array}{c}{\rm{\Delta }}{\omega }_{N}^{{B}_{1g}\mathrm{(1)}}(T)(-{\omega }_{0})(\frac{m}{2})\approx [\frac{{\partial }^{2}{J}_{1}^{xz}}{\partial {u}_{O1}^{2}}+4\frac{{\partial }^{2}{J}_{3}^{xz}}{\partial {u}_{O1}^{2}}({\langle {\tau }_{i}{\tau }_{j}\rangle }_{xz}+\delta {S}_{xz})]{(\frac{M(T)}{4{\mu }_{B}})}^{2}\\ \quad \quad +4\frac{{\partial }^{2}{J}_{3}^{xz}}{\partial {u}_{O1}^{2}}{\langle {\tau }_{i}{\tau }_{j}\rangle }_{xz}\delta {S}_{xz}\end{array}$$

and the Δ*ω*(*T*) of the out-of-phase bending mode at $\sim 490\,c{m}^{-1}$:

4 $$\begin{array}{c}{\rm{\Delta }}{\omega }_{N}^{{A}_{g}\mathrm{(3)}}(T)(-{\omega }_{0})(\frac{m}{2})\approx \gamma [\frac{{\partial }^{2}{J}_{1}^{xz}}{\partial {u}_{O1}^{2}}-\frac{1}{2}\frac{{\partial }^{2}{J}_{1}^{y}}{\partial {u}_{O2}^{2}}+4(\frac{{\partial }^{2}{J}_{3}^{xz}}{\partial {u}_{O1}^{2}}+\frac{1}{2}\frac{{\partial }^{2}{J}_{3}^{y}}{\partial {u}_{O2}^{2}})({\langle {\tau }_{i}{\tau }_{j}\rangle }_{xz}+\delta {S}_{xz})]\\ \quad \quad {(\frac{M(T)}{4{\mu }_{B}})}^{2}+4\gamma (\frac{{\partial }^{2}{J}_{3}^{xz}}{\partial {u}_{O1}^{2}}+\frac{1}{2}\frac{{\partial }^{2}{J}_{3}^{y}}{\partial {u}_{O2}^{2}}){\langle {\tau }_{i}{\tau }_{j}\rangle }_{xz}\delta {S}_{xz}\end{array}$$

**Figure 4:**
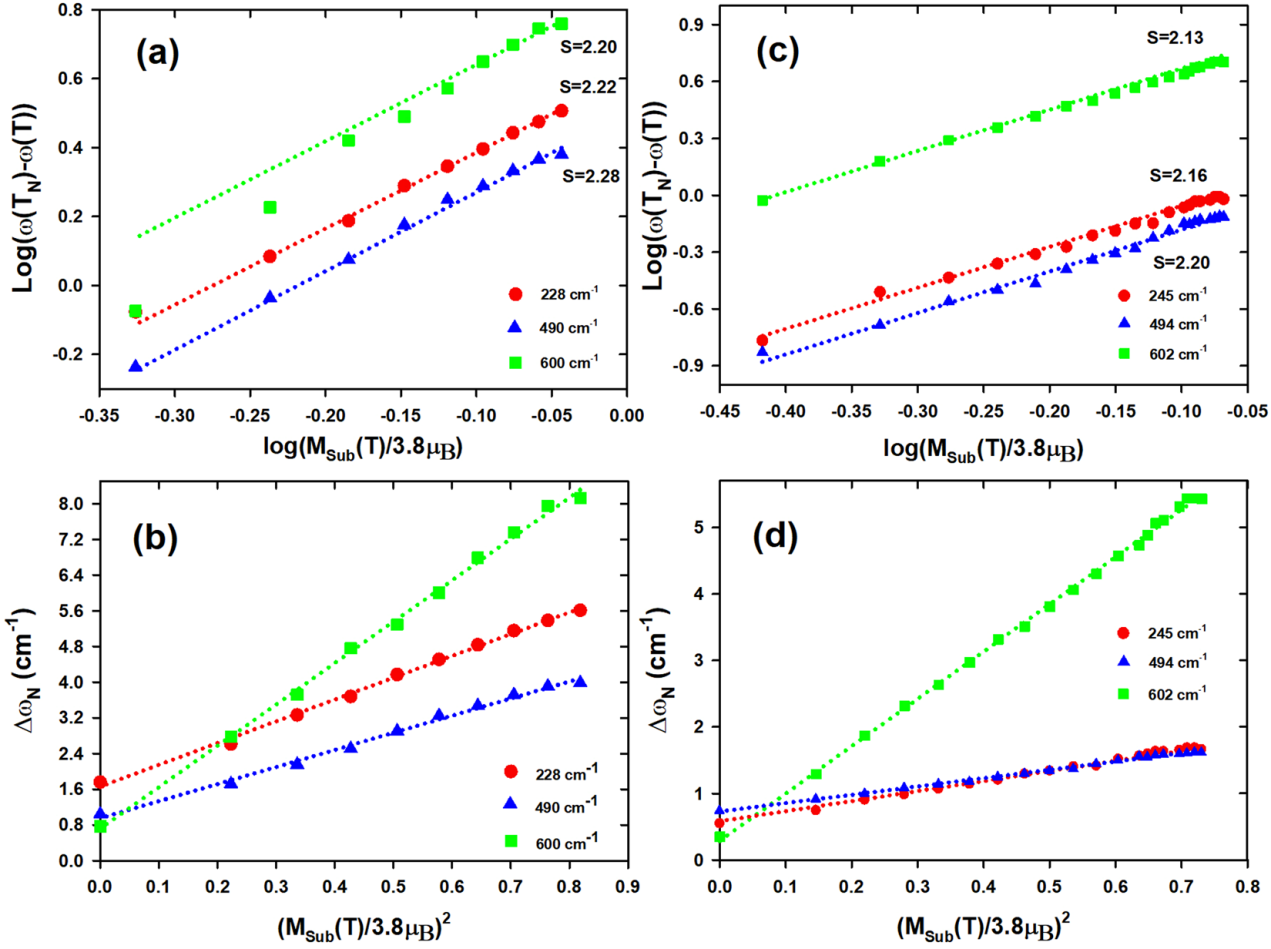
(**a**) A logarithmic plot of the mode frequency softening {*ω*(*T*_*N*_) − *ω*(*T*)} with respect to the sublattice magnetization for temperatures between 5 *K* and 100 *K* in *PrMnO*_3_. (**b**) Δ(*ω*) as a function of ${(\frac{{M}_{sub}(T)}{3.8{\mu }_{B}})}^{2}$ of the 228 *cm* ^−1^, 490 *cm* ^−1^ and 600 *cm* ^−1^ modes in *PrMnO*_3_. (**c**) and (**d**) represent their corresponding variations in *NdMnO*_3_. The variation of ${(\frac{{M}_{sub}(T)}{3.8{\mu }_{B}})}^{2}$ is taken from Refs^15,22,31^.

The common form of these last equations is a linear function *Y* = *SX* + *B* where *S* is the slope and *X* is the square of the sublattice magnetization. The intercept *B*, which is related to the effective force constant of orbital-spin-phonon coupling *k*_*OSP*_, is nonzero only if the interaction exists. This model is successfully used to explain the frequency softening of *LaMnO*_3_ phonons^27^. However, detailed studies and more concrete experimental evidences are needed to confirm that the *OSP* coupling clearly exists, below *T*_*N*_ in the *A*–type *RMnO*_3_ compounds. To assess the validity of the *OSP* coupling in the case of *PrMnO*_3_ (and *NdMnO*_3_), we have examined the frequency softening behavior, not only of the high frequency modes at 490 *cm* ^−1^ (494 *cm* ^−1^) and 600 *cm* ^−1^ (602 *cm* ^−1^), but also of the low frequency mode at 228 *cm* ^−1^ (245 *cm* ^−1^) since it corresponds to a rotational Raman-active mode sensitive to the orbital ordering fluctuations as observed in *KCuF*_3_^30^. The Δ*ω*(*T*) of this in-phase *MnO*_6_ *y* rotation mode can be written as:

5 $$\begin{array}{c}{\rm{\Delta }}{\omega }_{N}^{{A}_{g}\mathrm{(2)}}(T)(-{\omega }_{0})(\frac{m}{2})\approx [\frac{{\partial }^{2}{J}_{1}^{xz}}{\partial {u}_{RotO1}^{2}}+4\frac{{\partial }^{2}{J}_{3}^{xz}}{\partial {u}_{RotO1}^{2}}({\langle {\tau }_{i}{\tau }_{j}\rangle }_{xz}+\delta {S}_{xz})]{(\frac{M(T)}{4{\mu }_{B}})}^{2}\\ \quad \quad +4\frac{{\partial }^{2}{J}_{3}^{xz}}{\partial {u}_{RotO1}^{2}}{\langle {\tau }_{i}{\tau }_{j}\rangle }_{xz}\delta {S}_{xz}\end{array}$$

where *u*_*Rotk*_ is the displacement vector of the *k* ^*th*^ *O* ^2−^ ion relative to the rotational mode. According to Granado model (eq. 1), the variation of log(*ω*(*T*_*N*_) − *ω*(*T*)) versus $\mathrm{log}(\frac{{M}_{Sub}(T)}{3.8{\mu }_{B}})$ should yield a linear line with a slope of +2 if the spin-phonon coupling is solely responsible for the frequency softening below *T*_*N*_. Figure 4(a) (Fig. 4(c)) shows a logarithmic plot of the frequency softening {*ω*(*T*_*N*_) − *ω*(*T*)} in *PrMnO*_3_ (*NdMnO*_3_) for the 600 *cm* ^−1^ (602 *cm* ^−1^), 490 *cm* ^−1^ (494 *cm* ^−1^) and 228 *cm* ^−1^ (245 *cm* ^−1^) modes with respect to the sublattice magnetization $\mathrm{log}(\frac{{M}_{Sub}(T)}{3.8{\mu }_{B}})$ at temperatures between 5 *K* and ${T}_{N}\sim 100\,K$ (${T}_{N}\sim 80\,K$). The estimated slope *S* is significantly different from the prediction value of +2. The slope *S* for a linear fit (dashed line) is around 2.22 (2.16), 2.28 (2.20) and 2.20 (2.13) for the 228 *cm* ^−1^ (245 *cm* ^−1^), 490 *cm* ^−1^ (494 *cm* ^−1^) and 600 *cm* ^−1^ (602 *cm* ^−1^) modes respectively. This suggests that the spin-phonon coupling alone does not adequately explain the observed frequency softening below *T*_*N*_ in *PrMnO*_3_ and *NdMnO*_3_.

Figure 4(b) shows a good linear correlation between Δ*ω*(*T*) and $X={(\frac{{M}_{Sub}(T)}{3.8{\mu }_{B}})}^{2}$ which is in agreement with the linear function of equations 3, 4 and 5. The extrapolation of the linear function at *X* = 0 gives the intercept *B*: 1.65, 0.94 and 0.71 for the 228 *cm* ^−1^, 490 *cm* ^−1^ and 600 *cm* ^−1^ modes respectively. These behaviors are also observed in *NdMnO*_3_ with *B* values: 0.58, 0.73 and 0.29 for the 245 *cm* ^−1^, 494 *cm* ^−1^ and 602 *cm* ^−1^ modes respectively (Fig. 4(d). These finite positive values indicate that the spin and the orbital orders are also coupled in *PrMnO*_3_ and *NdMnO*_3_. In addition, the nonzero value of *B* at *T*_*N*_ implies that this coupling does not vanish completely above *T*_*N*_ but remains nonzero for a finite temperature range due to the magnetic correlations in the paramagnetic phase. The term *B* is interpreted as the effective force constant of the *OSP* coupling *k*_*OSP*_. The effective force constants *k*_*SP*_ (of spin-phonon coupling) and *k*_*OSP*_ (of orbital-spin-phonon coupling) are reported in Table 2. For the three modes, the *k*_*SP*_ and the *k*_*OSP*_ decrease with the ionic radius of the rare-earth. For all compounds, the *k*_*OSP*_ of the *A*_*g*_(3) ($\sim 490\,c{m}^{-1}$) mode is slightly stronger than those of the *A*_*g*_(2) and *B*_1*g*_(1) modes. This indicates that the *A*_*g*_(3) mode, intimately related to the Jahn-Teller distortions, is more sensitive to the spin-orbital coupling. The *k*_*OSP*_ of the *A*_*g*_(3) decreases with *r*_*R*_ (of *La*, *Pr* and *Nd*) while the ratio *k*_*OSP*_/*k*_*SP*_ is almost constant. These facts underline the important role of the ionic-radius size and the apical *O*2 vibration in the phonon modulation of the spin-orbital interaction in the *RMnO*_3_ *A*–type compounds.

**Table 2:** The effective force constants *k*_*SP*_ (spin-phonon coupling) and *k*_*OSP*_ (orbital-spin-phonon coupling) of the *A*_*g*_(2), *A*_*g*_(3) and *B*_1*g*_(1) Raman-active modes in *LaMnO*_3_^27^, *PrMnO*_3_ and *NdMnO*_3_.

Compounds/modes	*kSP* (dynes/cm)			*kOSP* (dynes/cm)			*kOSP*/*kSP* (%)		
Modes	*Ag*(2)	*Ag*(3)	*B*1*g*(1)	*Ag*(2)	*Ag*(3)	*B*1*g*(1)	*Ag*(2)	*Ag*(3)	*B*1*g*(1)
*LaMnO*_3_^27^	—	1000	—	—	70	—	—	7.0	—
*PrMnO*_3_	651	865	2622	54	66	61	8.2	7.6	2.3
*NdMnO*_3_	193	655	2413	20	51	25	10.3	7.7	1.0

As mentioned above, the frequency softenings of phonons in *TbMnO*_3_ and *DyMnO*_3_ are much weaker than those in *PrMnO*_3_ and *NdMnO*_3_. The origin of their frequency softening remains unclear: it has been qualitatively assigned to a spin-phonon coupling^32^ or to an expansion in the *Mn* − *O* bond lengths^22^. However, there are no concrete experimental proofs to determine their true origin. The phonon frequency shifts induced by the change of the ionic binding energies due to the lattice expansion/contraction, is usually approximated by the Grüneisen law Δ*ω*_*α*_(*T*)/*ω*_0_ = −*γ*_*α*_(Δ*V*/*V*_0_) where *γ*_*α*_ is the Grüneisen parameter for the normal mode *α*. This later approximation is applicable for isotopically expanded lattices. One should also consider the possibility of phonon frequency shifts due to lattice anomalies, even in the absence of a lattice unit cell volume change. Indeed, for some phonons the displacement of the involved atoms is either unidimensional or restricted in a plane. Blasco *et al*.^16^ have observed an expansion in *TbMnO*_3_ *a*–lattice parameter below 130 *K* and no significant changes are observed for the *b* and *c* parameters.

**Figure 5:**
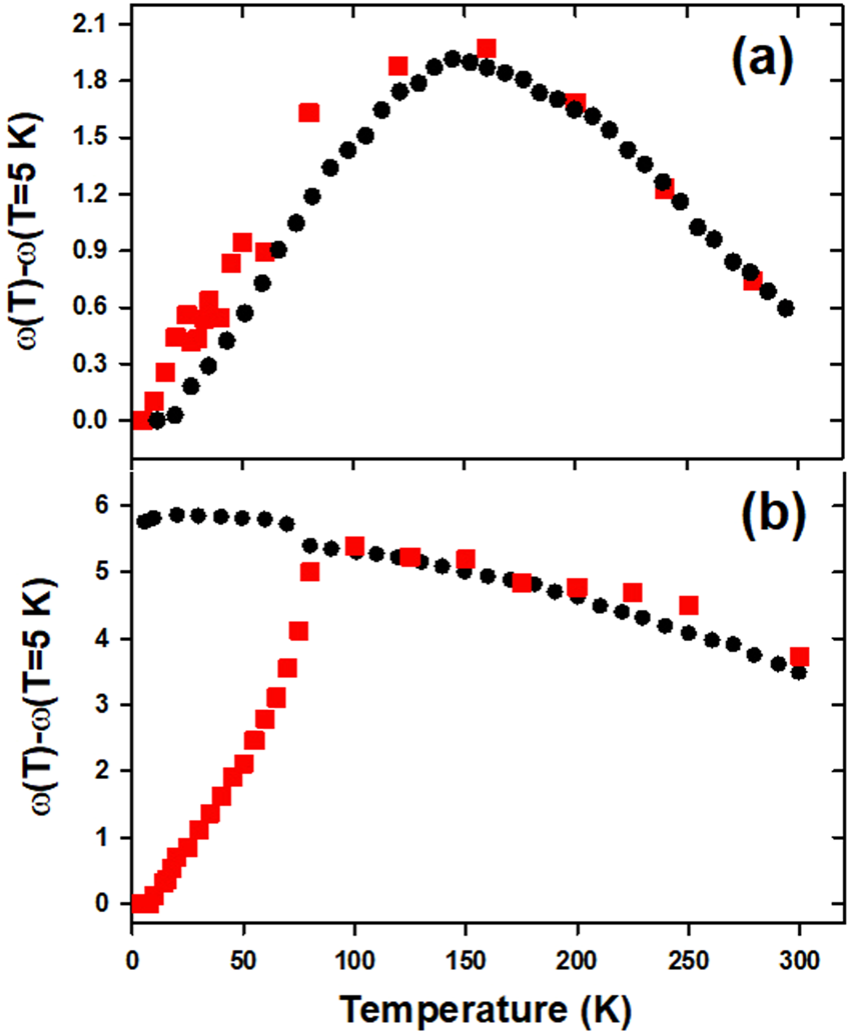
(**a**) The filled red squares show the temperature dependence of the 615 *cm* ^−1^ mode in *TbMnO*_3_. The filled black circles show the variation of −*γω*_0_Δ*A*/*A*_0_ as a function of temperature where *A* = *a* × *b* ^16^. In (**b**) their corresponding variations in *NdMnO*_3_.

Figure 5 shows the temperature dependence of the frequency of *B*_1*g*_(1) mode (red squares) in *TbMnO*_3_ (a) and *NdMnO*_3_ (b). The black dots indicate the variation of −*γω*_0_Δ*A*/*A*_0_ as a function of temperature where *A* = *a* × *b* and *γ* is the Grüneisen parameter. The frequency softenings of the *B*_1*g*_(1) mode are in agreement with the thermal expansion in the *ab*–plane with a Grüneisen parameter of $\sim 0.0416$ in the case of *TbMnO*_3_ whereas it is clearly not the case of *NdMnO*_3_ below $T\sim 80\,K$. This confirms that in the case of *TbMnO*_3_ the frequency shift of the *B*_1*g*_(1) mode is mainly due to an expansion in the *Mn* − *O* distances related to discrete orbital rearrangements close to the magnetic order transition, a phenomenon not discussed before, in this much-distorted compounds *RMnO*_3_ (*R* = *Tb* and *Dy*). Indeed, the negative thermal expansion, recently observed in some magnetic materials (*Ca*_2_ *Ru*_1−*x*_ *M*_*x*_ *O*_4_ where *M* = *Mn* and *Fe*) at low temperatures, is attributed to a strong coupling between orbital and magnetic orders^33,34^. In addition, the frustrated magnetic order in *TbMnO*_3_ is usually attributed to its frustrated orbital ordering as compared to *TbMnO*_3_^3^.

### Analysis of magnetic-field effects

In order to further investigate the spin-lattice coupling in a frustrated multiferroic system *TbMnO*_3_ againt a non-multiferroic compound *PrMnO*_3_, we also study the evolutions of Raman active phonon frequencies of both compounds under an applied magnetic field at 4.2 *K*, below ${T}_{N}^{R=Pr,Tb}$.

**Figure 6:**
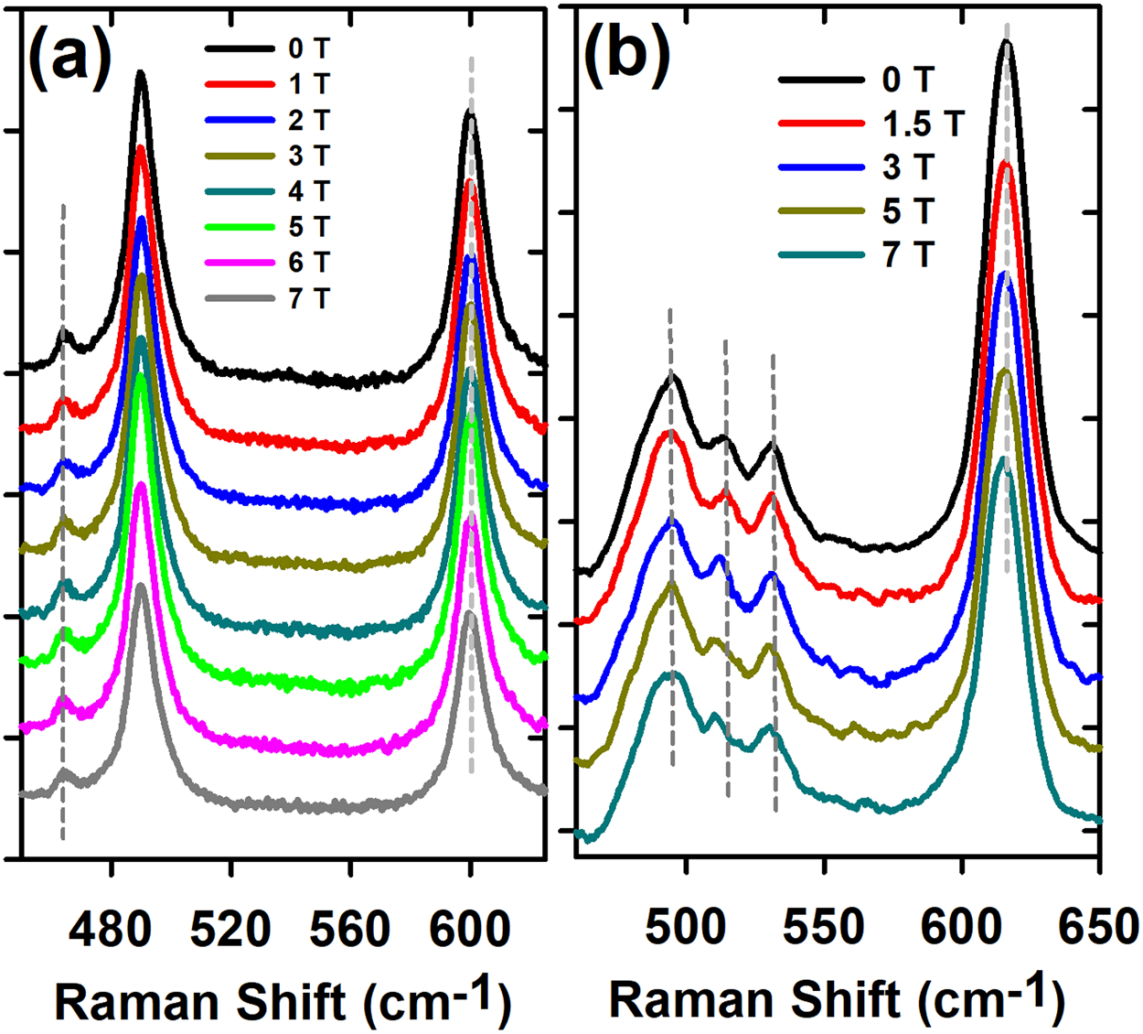
Unpolarised Raman spectra of *PrMnO*_3_ (**a**) and *TbMnO*_3_ (**b**) as a function of magnetic field (up to 7 T) applied along the *c*–axis.

Figure 6 shows the magnetic field dependence of the unpolarized macro-Raman spectra of *PrMnO*_3_ (a) and *TbMnO*_3_ (b) up to 7 Tesla. The applied magnetic field is quasi-parallel to the *c*–axis. While some of *TbMnO*_3_ phonon frequencies are shifted as a function of magnetic field, those of *PrMnO*_3_ remain unaffected. Similarly to *TbMn*_2_ *O*_5_^35^, these magnetic-field induced frequency shifts reflect the frustration of the spin configuration in *TbMnO*_3_ and its sensitivity to the presence of a magnetic field. The most affected phonons in *TbMnO*_3_, by the magnetic field, are the Jahn-Teller modes at 615 *cm* ^−1^ (*B*_1*g*_(1)) and 513 *cm* ^−1^ (*A*_*g*_(3)). Their frequencies soften by $\sim 1\,c{m}^{-1}$ and $\sim 2\,c{m}^{-1}$ at 7 Tesla respectively. These phonons involve mainly the *O*1 oxygen vibrations in the *xz*–plane and the *Mn* − *O* distances suggesting a magnetic-field modulation of the *Mn* − *O* − *Mn* bond lengths. These findings are in agreement with previous results indicating that a magnetic field induces a magnetoelastic coupling in *TbMnO*_3_^36,37^. Also, in our study of the *Tb* ^3+^ crystal-field excitations in *TbMnO*_3_ single crystals, we have found that some excitations are significantly shifted under an applied magnetic field along the *c*–axis at 4.2 *K* ^38^. This observation is in favor of oxygen displacements under magnetic-field in agreement with a prominent role of the oxygen ions in the multiferroicity of *TbMnO*_3_ and *DyMnO*_3_ as recently reported by Huang *et al*.^39^. Hence the ferroelectricity is related to local oxygen arrangements following the increase of the *Mn* − *O*1 bond length as induced by Dzyaloshinskii-Moriya interaction rather than a pure electronic mechanism as suggested by Kastura *et al*.^40,41^.

## Conclusion

In this comparative Raman study of the *PrMnO*_3_, *NdMnO*_3_, *TbMnO*_3_ and *DyMnO*_3_ single crystals, the phonon frequency shifts in *PrMnO*_3_ and *NdMnO*_3_ observed below *T*_*N*_, are explained by an orbital-spin-phonon coupling whereas those in *TbMnO*_3_ and *DyMnO*_3_, observed below 130 *K*, are attributed to an expansion of the *Mn* − *O* distances. In our *PrMnO*_3_ and *TbMnO*_3_ magneto-Raman measurements, it is shown that some *TbMnO*_3_ phonons are shifted as a function of magnetic field, while those of *PrMnO*_3_ remain unaffected. The magnetic-field dependence of the *TbMnO*_3_ phonon frequencies is associated to a magnetic modulation of the *O*1 oxygen displacements and suggests that *Mn* − *O*1 bond polarization may play a significant role in the magnetoelectric properties of *TbMnO*_3_.

## Methods

*RMnO*_3_ (*R* = *Pr*, *Nd*, *Dy* and *Tb*) single-crystals were grown by the floating zone method as described in reference^42^. The Raman spectra were recorded at temperatures between 300 *K* and 5 *K* and were obtained in the backscattering configuration using a *He* − *Ne* laser (632.8 *nm*) and a Labram-800 micro-Raman spectrometer equipped with a *X* − 50 objective microscope (focus diameter around $\sim 3\,\mu m$), an appropriate notch filter and a nitrogen-cooled *CCD* detector. The studied single crystals were mounted in a continuous flow temperature regulated liquid helium Janis cryostat. We have also measured the Raman active excitations under applied magnetic field up to 7 Tesla. The Raman measurements under magnetic field were obtained with an *Ar* ^+^ incident laser (514.5 *nm*) and with a magnetic field parallel to the *c*–axis.

### Data Availability

Most data generated or analysed during this study are included in this published article. The Raman spectra of *RMnO*_3_ (*R* = *Pr*, *Nd*, *Dy* and *Tb*) compounds at different temperatures are available from the corresponding author on reasonable request.
